# Mycorrhizal structures in mycoheterotrophic *Thismia* spp. (Thismiaceae): functional and evolutionary interpretations

**DOI:** 10.1007/s00572-022-01076-3

**Published:** 2022-04-14

**Authors:** Benjamin Feller, Martin Dančák, Michal Hroneš, Michal Sochor, Kenji Suetsugu, Stephan Imhof

**Affiliations:** 1 Fachbereich Biologie, Biodiversität der Pflanzen, Philipps-Universität, 35032 Marburg, Germany; 2 Department of Ecology and Environmental Sciences, Faculty of Science, Palacký University, Šlechtitelů 27, 78371 Olomouc, Czech Republic; 3 Department of Botany, Faculty of Science, Palacký University, Šlechtitelů 27, 78371 Olomouc, Czech Republic; 4 Centre of the Region Haná for Biotechnological and Agricultural Research, Crop Research Institute, Šlechtitelů 29, 78371 Olomouc, Czech Republic; 5 Department of Biology, Graduate School of Science, Kobe University, Kobe, Hyogo 657-8501 Japan

**Keywords:** *Thismia*, Mycoheterotrophy, Arbuscular mycorrhiza, Hyphal shape, Colonization pattern, Evolutionary progression

## Abstract

Achlorophyllous, mycoheterotrophic plants often have an elaborate mycorrhizal colonization pattern, allowing a sustained benefit from external fungal root penetrations. The present study reveals the root anatomy and mycorrhizal pattern of eight mycoheterotrophic *Thismia* spp. (Thismiaceae), all of which show separate tissue compartments segregating different hyphal shapes of the mycorrhizal colonization, as there are intact straight, coiled and peculiarly knotted hyphae as well as degenerated clumps of hyphal material. Those tissue compartments in *Thismia* roots potentially comprise exo-, meso- and endoepidermae, and exo-, meso- and endocortices, although not all species develop all these root layers. Differences in details among species according to anatomy (number of root layers, cell sizes and shapes) and colonization pattern (hyphal shapes within cells) are striking and can be discussed as an evolutionary series towards increasing mycorrhizal complexity which roughly parallels the recently established phylogeny of *Thismia*. We suggest functional explanations for why the distinct elements of the associations can contribute to the mycorrhizal advantage for the plants and, thus, we emphasize the relevance of structural traits for mycorrhizae.

**Supplementary information:**

The online version contains supplementary material available at 10.1007/s00572-022-01076-3.

## Introduction

*Thismia* spp. (Thismiaceae), rarely reaching 10 cm in height, are fully mycoheterotrophic plants (MHP, Leake 1994), entirely dependent on their mycorrhizal fungus not only for water and nutrients but also for 100% of their carbon supply (e.g. Merckx 2013). Knowledge of *Thismia* biodiversity has increased tremendously within the last 10 years. Within the 164 years between Griffith (1844) and Chantanaorrapint (2008), 47 species were described as new to science, and that number of species has more than doubled between Chiang and Hsieh (2011) and Siti-Munirah and Dome (2022). To date, *Thismia* comprises 96 species (Imhof 2010a onwards). The vast majority occurs in Southeast Asia, 15 originate from the New World tropics between Costa Rica and southern Brazil, and additionally, there is a remarkable extratropical location of *Thismia americana* in the vicinity of Chicago (Pfeiffer 1914, today extinct). Eight *Thismia* spp. were found in Australia, Tasmania and New Zealand, and three are known from Japan (data assembled from Imhof 2010a onwards).

Despite their small size, *Thismia* spp. captivate by their amazing diversity in flower shape and colour (e.g. Nuraliev et al. 2020). These highly diverse floral traits have been used for tribal or generic systematics initiated by Schlechter (1921, as a generic subdivision of the tribe Thismieae), advanced by Jonker (1938) and more recently reviewed in Kumar et al. (2017). However, Shepeleva et al. (2020) revealed that traditional systematics does not always reflect molecular-based phylogeny. They also showed that some morphological traits (e.g. staminal features and root morphology) seem to reflect phylogeny more than others (Shepeleva et al. 2020). Therefore, the diverse root systems of *Thismia*, including vermiform, coralloid and tuberous/filiform structures, are the subject of this study.

Due to the essential dependency on a well-organized mycorrhiza, roots (or rhizomes) are the critical components for MHP. There are three potentially contradicting requirements to the mycorrhizal organ of a MHP: (1) it should have plenty of primary tissue providing space for the nourishing hyphae, (2) it should have sufficient epidermal surface to increase the probability to become colonized by an appropriate fungus, and (3) transportation distance of fungus-derived carbon and nutrients should be either short or particularly secured. In contrast to green plants, which can renew lost root segments through photosynthetically assimilated carbon, a root disconnection in a MHP (e.g. through pathogens, vessel collapse, disruption by animals) means energy cut-off. Short and thick roots meet requirements (1) and (3) but fail to satisfy (2). On the other hand, long and thin roots achieve necessities (1) and (2) but are vulnerable with respect to requirement (3). This ‘mycoheterotroph’s dilemma’ (Imhof 2010b) has been overcome in various ways, either by a particularly strong tertiary endodermis in long and thin roots (e.g. *Dictyostega orobanchoides*, Imhof 2001); an intermediate state of root thickness and length (e.g. *Voyria truncata*, Imhof and Weber 1997); a sophisticated mycorrhizal pattern, allowing a sustained benefit from the few fungal penetrations acquired by short and thick roots (e.g. *Voyria tenella*, Imhof 1997); or a filiform root extension on a tuberous root base combined with a complex fungal colonization pattern (*Afrothismia* spp., Imhof et al. 2020). The present study aims to reveal the mycorrhizal pattern in the three root types of *Thismia* spp. (vermiform, coralloid and tuberous) and the functional relevance of the anatomical and hyphal structures found. The results also may elucidate *Thismia* phylogeny and evolution.

## Materials and methods

We investigated fragments of subterranean organs of eight *Thismia* species from Panama, Malaysia and Japan (for collecting data and number of studied specimens see Supplementary material) stored in 70% ethanol, with regard to their morphology, anatomy and mycorrhizal pattern. Due to the rarity of the species, only a few individuals per species could be analysed. However, the interspecific congruency of the presented data as well as the results of an additional six *Thismia* spp. from previous studies indicates very low intraspecific variation such that our general conclusions should not be affected.

The root fragments were investigated for their external morphology, photographed with a Moticam 2300 digital camera device (Motic) mounted on a Leica S6D stereo microscope (Leica Instruments) and then prepared for anatomical studies. After dehydration in an ascending ethanol series, roots and root/shoot system were embedded in Unicryl™ (British Biocell Int.). Serial sections of 3–4 µm were made (Leica 2065 Supercut, glass knives prepared by LKB 2078 Histo Knifemaker, LKB Produkter AB, Research Instruments), stained with toluidine blue (1 g toluidine blue O + 1 g sodium tetraborate in 100 ml distilled H_2_O, after Harris in Krause 1927) and mounted in Corbit-Balsam. Histological tests were performed on paraffin-embedded material (Merck, melting point 56–58 °C) sectioned with a Leitz 1512 hand microtome (Leica Instruments) and deparaffinized with Xylol. Suberin was visualized using the Oil Red O supersaturated isopropanol technique diluted to 60% with distilled water after Lillie (1944); starch and lignin were tested by the iodine test and Phloroglucine/HCl, respectively (Jensen 1962). Anatomical investigations were performed using a Leitz DMRB-Microscope (Leica Instruments), equipped with a Moticam 2300 digital camera.

Samples for confocal laser scanning microscopy (CLSM) were prepared after the protocol explained in Rath et al. (2014), using Calcofluor White M2R (a non-specific stain that enhances autofluorescence) and WGA (wheat germ agglutinin) conjugated with Alexa Fluor^®^ 633 (fungus specific) as fluorochromes. The samples were excited with a Leica TCX SP5 confocal laser scanning microscope (Leica Instruments) using an UV-Laser (405 nm, for Calcofluor) and a HeNe-Laser (633 nm, for Alexa Fluor^®^). The resulting image stacks were analysed and visualized with AMIRA^®^ (FEI^®^ Visualization Sciences Group, Düsseldorf) and Leica Confocal Software (LCS, Leica Microsystems).

### Terminological aspects

The mycorrhizal terminology used in classical studies of *Thismia* spp. is incongruent for comparisons. For example, authors used topological (e.g. ‘subepidermal layer’, Pfeiffer 1914, ‘exocortex’, McLennan 1958) or functional terms (‘sheath’, ‘limiting layer’, Groom 1895) to address distinct root layers. In order to comprehend the comparison between our results and the structures reported by different authors, we needed to harmonize the heterogeneous terminology used for *T. aseroe* (Groom 1895), *T. javanica* (Janse 1896; Meyer 1909; Bernard and Ernst 1910), *T. clandestina*, *T. versteegii* (Bernard and Ernst 1911; Jonker 1938 synonymized *T. versteegii* with *T. crocea*, Larsen 1965 opposed this view), *T. americana* (Pfeiffer 1914) and *T. rodwayi* (Coleman 1936; McLennan 1958) based on the following reasoning. The common feature of all *Thismia* roots investigated so far is the cell layer hosting coarse coils of non-degenerating hyphae (see the “Results” section). This layer is the second root layer in *T. javanica* (Bernard and Ernst 1910) and *T. rodwayi* (Coleman 1936; McLennan 1958), consequently called ‘subepidermal layer’ or ‘exocortex’, respectively, by the authors. In *T. clandestina* and *T. versteegii* (Bernard and Ernst 1911) this particular layer appears as the fourth to fifth root layer, and the authors use ‘Pilzwirtzellen’ (= fungus host cells) for it, after Magnus (1900), who coined this term for *Neottia nidus-avis* (Orchidaceae). In contrast, in *T. abei* (this study), this layer is the epidermis. In order to realign the differing terminology, we use this characteristic cell layer as basis for a combined topological and functional terminology. Because it is topologically as well as genealogically the epidermis in *Thismia abei* (see Fig. 2D), all additional cell layers outside of it in other *Thismia* spp. should also belong to the epidermis and be differentiated as exo-, meso- and endoepidermis. Similarly, we distinguish between exo-, meso- and endocortex, parts of which have been called ‘limiting layer’ and ‘mediocortex’ (Groom 1895), or simply ‘cortex’ (Pfeiffer 1914; McLennan 1958).

## Results

**Fig. 1:**
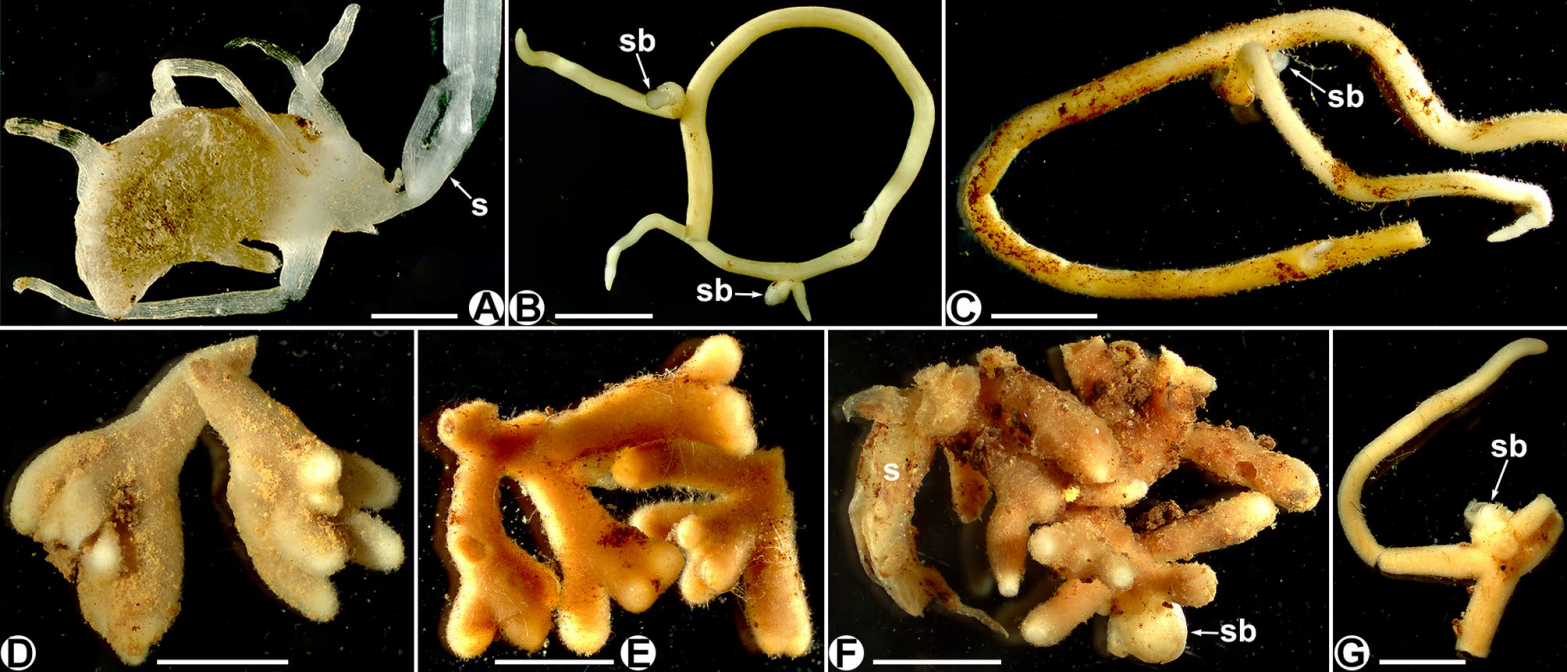
Subterranean organs of *Thismia* spp. **A** *T. luetzelburgii* (tuberous), **B** *T. abei* (vermiform), **C** *T. minutissima* (vermif orm), **D** *T. brunneomitra* (coralloid), **E** *T. goodii* (coralloid), **F** *T. viridistriata* (coralloid), **G** *T. neptunis* (vermiform). s = shoot, sb = shoot bud. Scale bars = 3 mm

Our study distinguishes three main morphological root types and six morpho-anatomical mycorrhizal patterns among studied *Thismia* spp. The subterranean organs of *Thismia luetzelburgii* and *T. panamensis* are almost identical. The shoot arises from a globose to somehow elongated tuberous root (4–11 × 3–6 mm in *T. luetzelburgii*, 3.5–15 × 2–5 mm in *T. panamensis*, Maas et al. 1986) with filiform roots radiating from the tuber (Fig. 1). *Thismia brunneomitra*, *T. goodii* and *T. viridistriata* have coralloid root systems, with abbreviated and densely branched roots. *Thismia abei*, *T. minutissima* and *T. neptunis* show vermiform roots, having few ramifications. They develop shoot buds in the axils of the root ramifications. Roots of the non-tuberous *Thismia* spp. measure between 0.8 and 1.8 mm in diameter. Root hairs are present in *T. brunneomitra*, *T. goodii* and *T. minutissima*, they are very short in *T. viridistriata* and *T. neptunis*, and absent in *T. abei*, *T. luetzelburgii* and *T. panamensis* (see Fig. 1).

**Fig. 2:**
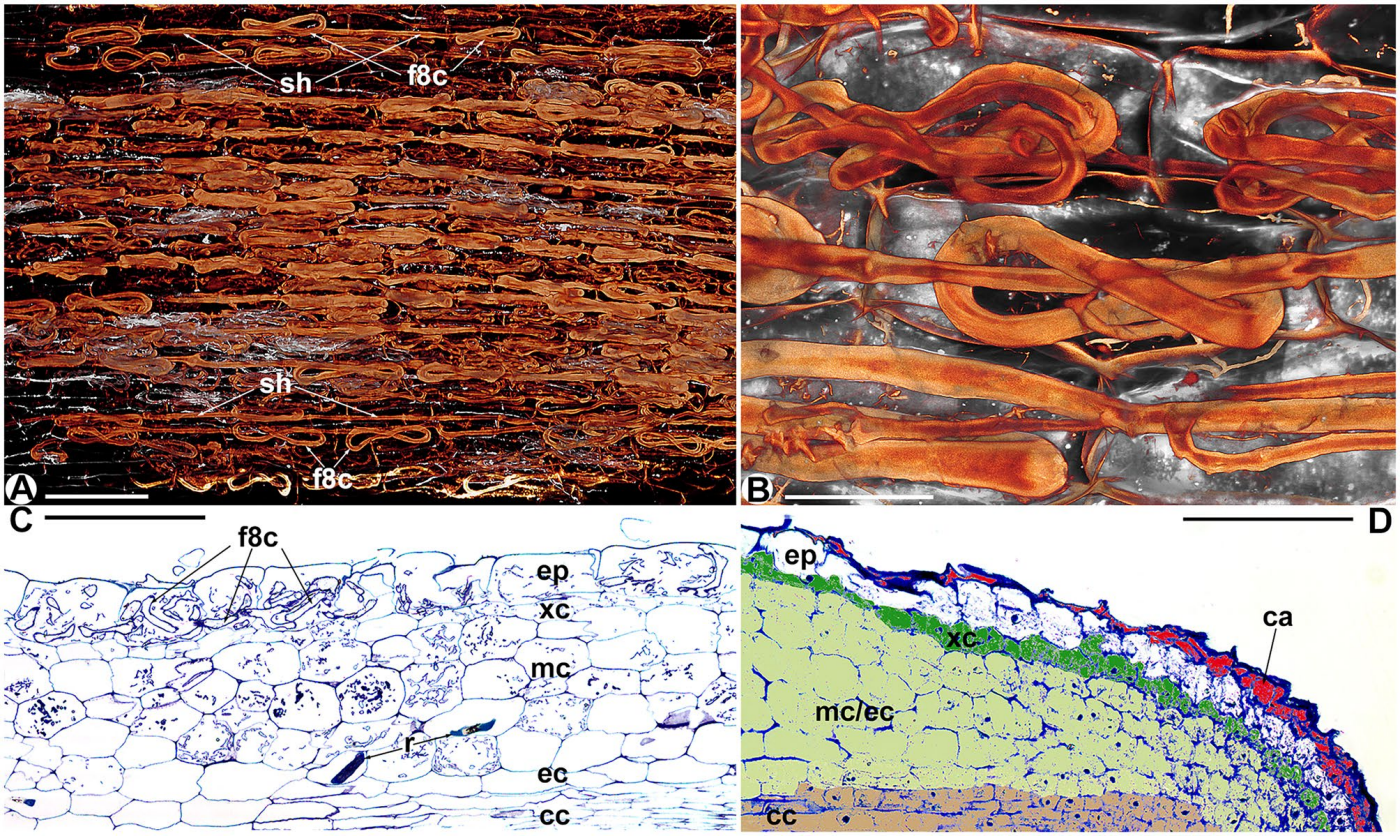
Roots of *Thismia abei*. **A** Projection of several optical layers through the epidermis, showing straight-growing hyphae (sh) and figure-of-eight coiled hyphae (f8c). Scale bar = 200 µm. **B** Close-up of a figure-of-eight coil. Scale bar = 50 µm. **C** Longitudinal section showing intact figure-of-eight hyphal coils (f8c) in the epidermis (ep), smaller cells in the exocortex (xc), degenerating hyphal coils in the mesocortex (mc) and a fungus-free endocortex (ec). r = raphid bundles, cc = central cylinder, scale bar = 200 µm. **D** Longitudinal section of a root tip, the colours indicate the derivatives of distinct meristem initials. ca = calyptra, ep = epidermis, xc = exocortex, mc/ec = mesocortex/endocortex, cc = central cylinder, scale bar = 100 µm

**Fig. 3:**
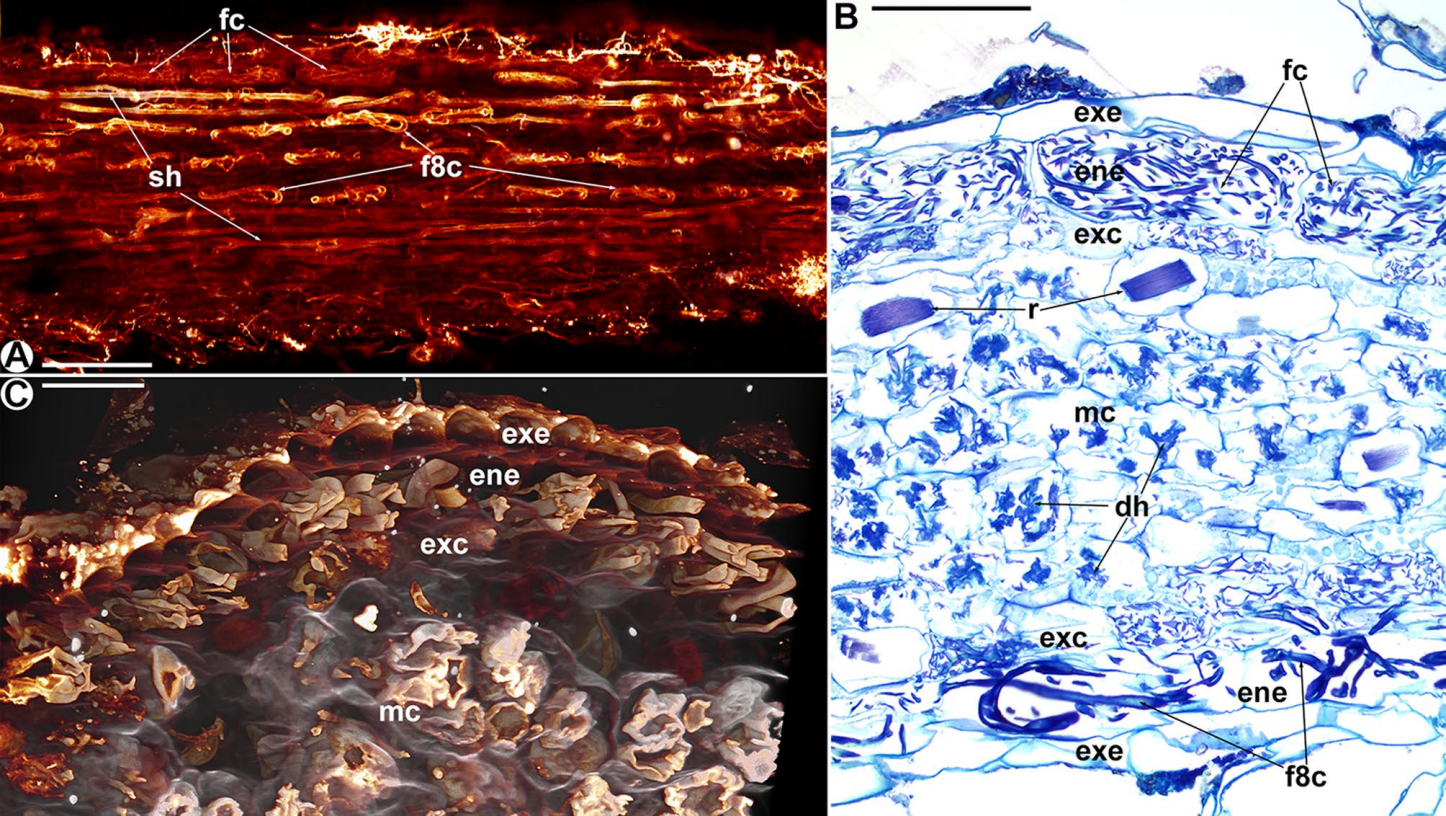
Roots of *Thismia minutissima*. **A** Projection of several optical layers through the endoepidermis, showing straight-growing hyphae (sh), coarse figure-of-eight hyphal coils (f8c) and coils of fine hyphae (fc). Scale bar = 200 µm. **B** Longitudinal section showing the fungus-free exoepidermis (exe), the endoepidermis (ene) with intact fine (fc) and figure-of-eight hyphal coils (f8c), hyphal coils with intermediate signs of degeneration in the smaller cells of the exocortex (exc), and degenerated hyphal coils (dh) in the mesocortex (mc). r = raphid bundles, scale bar = 100 µm. **C** Confocal laser scan micrograph (cross-view) giving a three-dimensional aspect of the root layers exoepidermis (exe), endoepidermis (ene), exocortex (exc) and mesocortex (mc). Scale bar = 50 µm

**Fig. 4:**
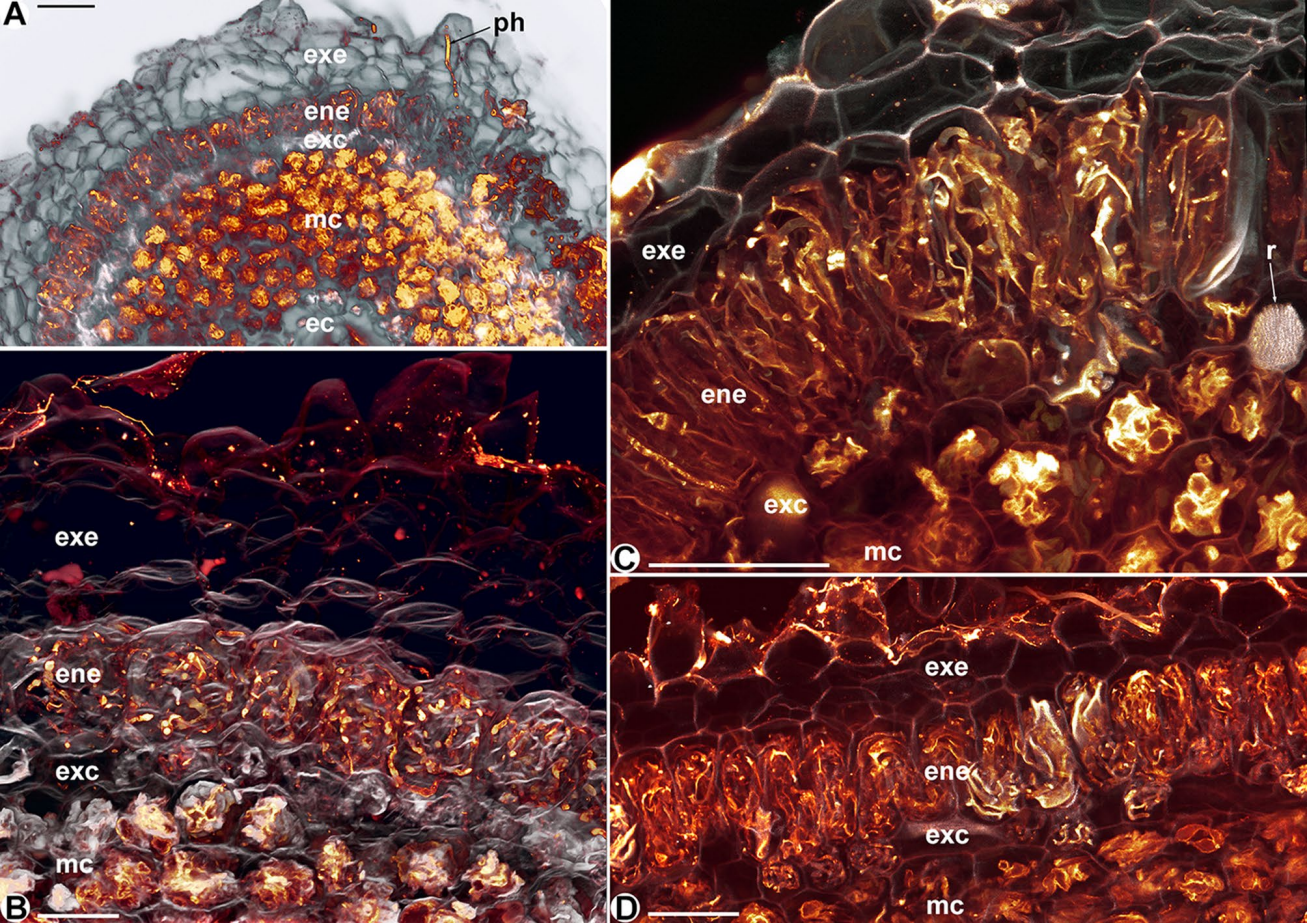
Confocal laser scan micrographs of roots of *Thismia brunneomitra* **A, B** and *Thismia goodii* **C, D**. **A** Cross-view showing the multilayered, fungus-free exoepidermis (exe), the isodiametrically enlarged cells of the endoepidermis (ene), the anatomically distinct exocortex (exc), the fungus digesting mesocortex (mc) and a fungus-free endocortex (ec). An externally penetrating hypha (ph) crosses the exoepidermis, heading for the endoepidermis. Scale bar = 100 µm. **B** Longitudinal view and close-up of the structures explained in Fig. 4A. Scale bar = 50 µm. **C** Cross-view showing the two-layered exoepidermis (exe), the radially enlarged cells of the endoepidermis (ene), the smaller and intermediate digestive cells of the exocortex (exc), and the fungus digesting mesocortex (mc). r = raphid bundle, scale bar = 100 µm. **D** Longitudinal view of the structures explained in Fig. 4C. Scale bar = 100 µm

In longitudinal and cross-sections, the roots of the non-tuberous *Thismia* spp. have the following common features. There is always an epidermal layer containing up to 12-µm thick, mostly coiled hyphae, which stay intact compared to the fungal material in the mesocortex (e.g. Figs. 2 and 3). The multilayered mesocortex predominantly shows amorphous clumps of fungal material derived from hyphal coils. The innermost zero to three endocortex layers stay free of hyphae (e.g. Fig. 2C and 4A). The layer between the epidermis and mesocortex, the exocortex, contains hyphae with intermediate signs of degeneration (e.g. Figs. 3C and 4D). It consists of tendentiously smaller cells than epidermis and mesocortex in *Thismia abei*, *T. minutissima*, *T. brunneomitra* and *T. goodii* (e.g. Figs. 3B and 4). There is no starch in *Thismia* root cells, although reactions to iodine solution, mapping degenerated hyphae based on concentrated chitin, could be misinterpreted as such. Lignin was noticed only in the wall fortifications of the xylem vessels in the central cylinder, and suberin, except for Casparian strips in some endodermae, was not found. Fungal colonization is always intracellular, and arbuscules were never seen. Vesicles were seen in the filiform root parts of *T. luetzelburgii* and *T. panamensis*, the roots of *T. abei* and the mesoepidermis of *T. neptunis*. Raphides may occur anywhere in the roots; cells with raphides were never seen to contain hyphae (e.g. Figs. 3B and 4C). A schematic comparison of the following specific mycorrhizal patterns is shown in Fig. 7; their traits in the Old World *Thismia* spp. are summarized in Table 1.

**Fig. 5:**
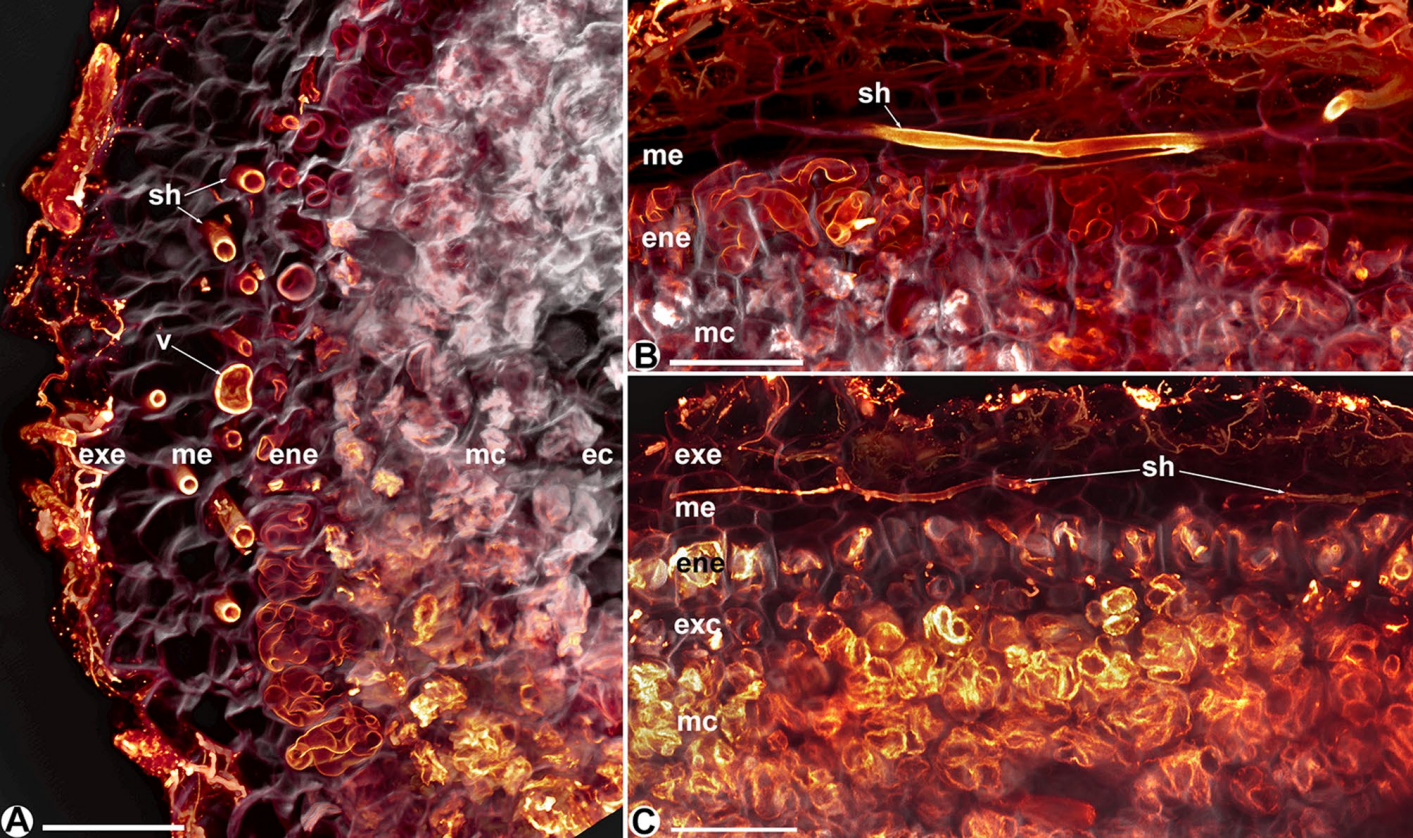
**A** Confocal laser scan micrographs of a cross-view of a root of *Thismia neptunis*, showing the fungus-free exoepidermis (exe), the mesoepidermis (me) with intact straight-growing hyphae (sh) and one of the rare vesicles (v), the endoepidermis with intact coiled hyphae, the mesocortex (mc) with degenerated clumps of hyphal material and a fungus-free endocortex (ec). Scale bar = 100 µm. **B** Longitudinal view of the structures explained in Fig. 5A. Scale bar = 100 µm. **C** Confocal laser scan micrograph of a longitudinal view of a root of *Thismia viridistriata*. Explanation see Fig. 5A. In contrast to Fig. 5A, B, the exocortex (**exc**) is faintly visible. Scale bar = 100 µm

**Table 1:** Comparisons among root anatomical and mycorrhizal components in Old World *Thismia* spp. investigated in this study

	*T. abei*	*T. minutissima*	*T. brunneomitra*	*T. goodii*	*T. viridistriata*	*T. neptunis*
Root morphology	Vermiform, root hairs absent	Vermiform, root hairs present	Coralloid, root hairs present	Coralloid, root hairs present	Coralloid, root hairs present	Vermiform, root hairs present
Exoepidermis	Absent	1-layered, without hyphae	3–4-layered, without hyphae	2-layered, without hyphae	1-layered, without hyphae	1-layered, without hyphae
Mesoepidermis	Absent	Absent	Absent	Absent	2-layered, with straight-growing hyphae	2-layered, with straight-growing hyphae
Endoepidermis (epidermis in *T. abei*)	With persistent coiled and straight-growing hyphae	With persistent coiled and straight-growing hyphae	Cells isodiametrically enlarged, with persistent coiled hyphae	Cells radially enlarged, with persistent coiled hyphae	With persistent coiled hyphae	With persistent coiled hyphae
Exocortex	Cells smaller than in neighbouring layers, with intermediate persistent, coiled hyphae	Cells smaller than in neighbouring layers, with intermediate persistent, coiled hyphae	Cells smaller than in neighbouring layers, with intermediate persistent, coiled hyphae	Cells smaller than in neighbouring layers, with intermediate persistent, coiled hyphae	With intermediate persistent, coiled hyphae	With intermediate persistent coiled hyphae
Mesocortex	Amorphous fungal material	Amorphous fungal material	Amorphous fungal material	Amorphous fungal material	Amorphous fungal material	Amorphous fungal material
Endocortex	Without hyphae	Without hyphae	Without hyphae	Without hyphae	Without hyphae	Without hyphae

The epidermis of *Thismia abei* (Fig. 2, pattern A in Fig. 7) is single-layered and shows both coiled and straight-growing hyphae within its cells, often in a regularly alternating manner (Fig. 2A). The coils often attain a characteristic shape, resembling a ‘figure of eight’ knot (Fig. 2B). Its exocortex is made of smaller cells than in the epidermis and mesocortex (Fig. 2C). Examination of the root apical meristem revealed the origin of the epidermis from its own apical initial, following the calyptrogen, whose descendants, as expected, cease shortly behind the root tip. The exocortex has its own initial, too, or may be part of the periblem following proximally. The plerome is diffuse (Fig. 2D).

*Thismia minutissima* (Fig. 3, pattern B in Fig. 7) has a two-layered epidermis. The endoepidermis contains intact coiled or straight-growing hyphae; the exoepidermis is free of hyphae. In contrast to *T. abei*, coils in the endoepidermis can be built up not only of coarse but also fine hyphae resulting in delicate coils (Fig. 3A, B). The coarse coils are reminiscent of the ‘figure of eight’ knots as in *T. abei* (Fig. 3A).

*Thismia brunneomitra* (Fig. 4A, B, pattern C1 in Fig. 7) develops 3(–4) exoepidermal fungus-free cell layers, which are traversed directly by newly penetrating hyphae from the rhizosphere until they reach the isodiametrically enlarged cells of the endoepidermis containing coiled hyphae only.

In contrast to the isodiametric endoepidermal cells of *T. brunneomitra*, those cells of *T. goodii* (Fig. 4C, D, pattern C2 in Fig. 7) are radially enlarged, in cross (Fig. 4C) as well as in longitudinal view (Fig. 4D), i.e. radial cell walls are longer than tangential and longitudinal ones. They also host coiled hyphae. The exoepidermis consists of only two layers.

*Thismia neptunis* (Fig. 5A, B) and *T. viridistriata* (Fig. 5C, pattern D in Fig. 7) are nearly identical in their root anatomy and mycorrhizal colonization pattern, although the former has coralloid and the latter vermiform roots. Both have a fungus-free exoepidermis, a two-layered mesoepidermis with straight-growing, non-degenerating hyphae in its cells, and an endoepidermis also with intact but coiled hyphae. An exocortex is not always clearly discernible.

**Fig. 6:**
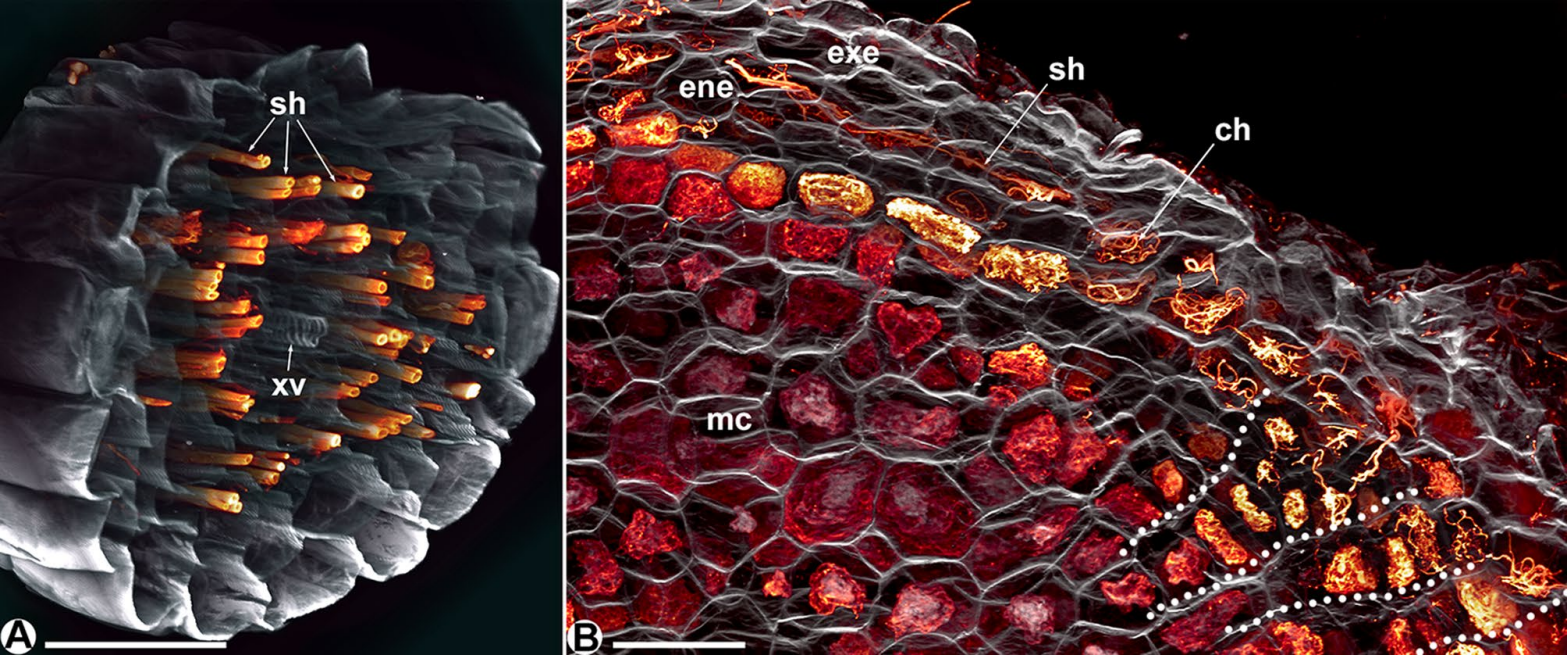
**A** Confocal laser scan micrograph of a filiform root of *Thismia luetzelburgii* in cross-view, hosting intact straight-growing hyphae (sh). xv = xylem vessel, scale bar = 100 µm. **B** Confocal laser scan micrograph of a tuber of *Thismia panamensis*, with a fungus-free exoepidermis (exe), an endoepidermis (ene) hosting intact coiled (ch) as well as straight-growing hyphae (sh) and a mesocortex (mc) with degenerating hyphal coils. The dotted lines mark presumably ontogenetic series of cells, indicating an ongoing growth of the tuber

**Fig. 7:**
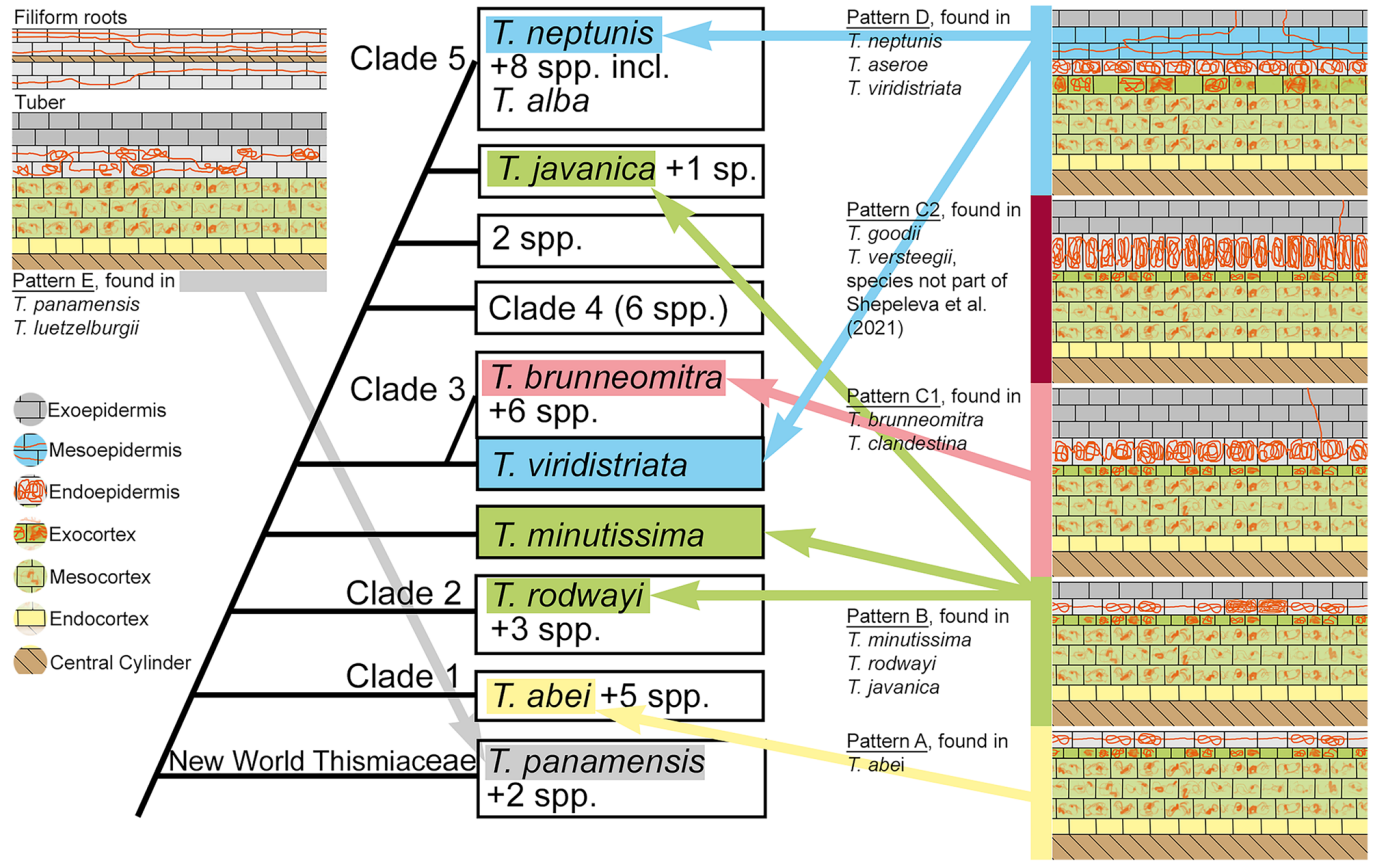
Schematic views of the mycorrhizal patterns in *Thismia* spp. and their appearance in the phylogenetic tree, simplified from Shepeleva et al. (2020)

The filiform roots of the tuberous *T. luetzelburgii* and *T. panamensis* (Fig. 6, pattern E in Fig. 7) only show intact straight-growing hyphae (Fig. 6A). Reaching the tuber, these hyphae colonize a double-layered endoepidermis; the likewise double-layered exoepidermis stays free of hyphae. However, the histological compartmentation is not as distinct as in the other *Thismia* spp. (Fig. 6B). In the endoepidermal cells, the hyphae may coil but also grow straight in tangential or longitudinal direction to distribute themselves around the tuber. There is no exocortex; instead, signs of hyphal degeneration seem to increase towards the inner tissue. Moreover, ladder-like radial cell rows indicate some cell division potential in the outer tissue of the tuber (dotted lines in Fig. 6B), possibly indicating ongoing primary growth of the tuber.

## Discussion

### Functional aspects

Although arbuscules are absent, the fungal associations in *Thismia* spp. are arbuscular mycorrhizae (AM) as indicated by morphological (e.g. Suetsugu and Ishida 2011, this study) and molecular evidence (Gomes et al. 2016; Merckx et al. 2017). The lack of arbuscules substantiates the experiences of the senior author on MHP mycorrhizas between Imhof and Weber (1997) and Imhof et al. (2020) who never found arbuscules in AM-dependent MHP, arguing against phenological reasons for the absence of arbuscules. Correspondingly, arbuscules, meant to provide the interface for active nutrient and carbon exchange between plant and fungus, are functionally obsolete when the entire fungal content of a cell becomes digested. Similar to other MHP taxa (e.g. Imhof 1997, 2003, 2007; Imhof et al. 2020), the mycorrhizal structures in *Thismia* spp. are complex and show distinct fungal shapes in separate root tissue compartments for specific functions, mainly for keeping hyphae intact in certain tissues and digesting them in others. The immediate benefit of this strategy is a sustained use of the presumably limited external penetrations by the fungus (because of the confined root surface) for the plant. Beyond that, specific shapes of hyphae can serve for specific assignments. Straight-growing hyphae, for example, are convenient for quickly spreading the fungal colonization along the root, certainly an important requirement for MHP with few penetration events. And in fact, several MHP implement straight-growing hyphae in their mycorrhizal pattern (e.g. Imhof 1997, 2007; Imhof et al. 2020), including *Thismia* spp. (this study). Yet, straight hyphae fail to comply with another necessity for MHP, namely, to constantly fuel the digesting tissue with numerous short-lived side branches for carbon and nutrient supply. The number of side branches for a given length of parent hypha supposedly is limited, simply because the branches, once developed, will block the development of new ones at that particular spot. Hence, straight-growing hyphae providing the conceivable minimum hyphal length per cell are inappropriate for this function, whereas if coiled, long segments of hyphae fit into a cell. This also elucidates the intact coils in other MHP, particularly the yet not understood spirally arranged hyphal loops around the root tubercle of *Afrothismia saingei* (Imhof 1999,1). Furthermore, the bigger the cell, the longer the coiled parent hyphae can be, and the more side branches for digestion may develop. This might explain the evolution of larger cells in the endoepidermae of *T. brunneomitra* (see Fig. 4A, B) and *T. clandestina* (see plate X/1 in Bernard and Ernst 1911). Besides, if a cell lumen is not evenly enlarged but predominantly increased in the radial direction, as is the case in *T. goodii* (see Fig. 4C, D) and *T. versteegii* (see plate XII/1 in Bernard and Ernst 1911), even more hyphal length fits along a given root length, which is directly advantageous for sending as much hyphae as possible into the digesting tissue for carbon uptake. Consequently, a MHP should have both straight and coiled intact hyphae. *Thismia abei*, *T. minutissima* and *T. rodwayi* (McLennan 1958) alternate both hyphal shapes in the epidermis or endoepidermis, thereby realizing a compromise between both necessities (i.e. spreading the fungal colonization and fueling the digesting tissue) within a limited tissue compartment. In terms of three-dimensional geometry, the ‘figure of eight’ knots in *T. abei* and *T. minutissima* are the longest possible segment of an up to 12-µm thick hypha in a relatively small, longitudinally elongated cell, using the full space capacity including the central gap of a hyphal loop. In *T. neptunis*, *T. viridistriata* and *T. aseroe* (Groom 1895), a compromise of necessities is not needed because straight-growing and coiled hyphae both have their distinct tissue compartments, the mesoepidermis and endoepidermis, respectively, thus separating large-scale (straight hyphae) and fine-scale (coiled hyphae) dispersal of the fungus within the root. *Thismia brunneomitra* and *T. goodii*, however, miss straight-growing hyphae, although a multilayered exoepidermis is present. These two species have coralloid root systems; their roots are abbreviated and densely clumped. Rapid longitudinal spread, which is desirable in vermiform roots, apparently is not required. Contradicting this explanation, *T. viridistriata* does have coralloid roots together with straight-growing hyphae in its mesoepidermis (see below for an evolutionary interpretation). *Thismia goodii* and *T. versteegii* show radially enlarged cells in the endoepidermis, which, as explained above, can potentially host long parent hyphae per root segment. The smaller cells observed in the exocortices of *T. abei*, *T. minutissima*, *T. brunneomitra* and *T. goodii* also have been reported in *T. rodwayi* (McLennan 1958) and *T. aseroe* (Groom 1895, called ‘limiting layer’) and were depicted in *T. americana* (see plate IIIV/7 in Pfeiffer 1914) and, therefore, seem to be not accidental. Exocortices appear to have an intermediate digestive power (less obvious in *T. neptunis* and *T. viridistriata*), possibly to support the distribution deeper into the cortex. The digestion process in the mesocortex supposedly is rather quick because we hardly find intact hyphae therein and the variable size of the fungus-free endocortex also indicates limited mesocortical spread. Hence, the side hyphae from the endoepidermis might degenerate too fast for further growth, if not conveyed by a less digestive exocortex. A cell volume reduction, thus, may be the result of economizing a tissue that neither is essential to provide parent hyphae length nor for digestion of fungal material.

In contrast to the above-mentioned *Thismia* spp. having stationary tissue compartmentation, the less differentiated compartments in the tubers of *T. luetzelburgii* and *T. panamensis* might indicate shifting compartmentation. That is, coils which once appear intact may later degenerate when newly developed endoepidermal cells can host newly arriving hyphae from the filiform roots. This also would explain the dimension of the tuber as well as the gradient of degeneration stages seen in some cell rows (see Fig. 6B). This fundamentally deviating mycorrhizal strategy underlines the distributional and phylogenetic gap between these New World versus the Old World *Thismia* spp. considered in this study.

### Evolutionary aspects

Because we know about the prepenetration apparatus (Genre 2005, 2008) through which a plant can form the shape of hyphae within a cell, mycorrhizal structures can be seen as a trait of the plant and are, together with the anatomy hosting them, subject to evolution. Indeed, the differences in *Thismia* root anatomy and mycorrhizal pattern can be arranged in order of increasing complexity, consistent with the current phylogeny of the genus (Shepeleva et al. 2020). *Thismia abei* has only one epidermal layer. In contrast, three other species develop an additional fungus-free exoepidermis: *T. minutissima*, *T. rodwayi* (McLennan 1958) and *T. javanica* (Bernard and Ernst 1910, Janse 1896, Meyer 1909, the last two authors erroneously named their material *T. clandestina*, realized and noted by Schlechter 1921). The exoepidermis may play a role in fungal recognition or segregation, or may protect the functionally essential endoepidermis. *Thismia goodii* has a two-layered exoepidermis, *T. clandestina* as well as *T. versteegii* (Bernard and Ernst 1911) two to three layers, and *T. brunneomitra* three to four exoepidermal layers. This multilayered exoepidermis eventually gains functional relevance as a mesoepidermis, when it hosts straight-growing hyphae in *T. neptunis*, *T. viridistriata* and *T. aseroe* (Groom 1895). Apart from these primary changes, the variations in cell sizes (endoepidermae, exocortices) may also help to support the mycorrhiza’s functionality (see Functional aspects). The neotropical representatives *T. luetzelburgii* and *T. panamensis*, although likewise showing intact straight-growing as well as coiled hyphae, fungus-free exoepidermae and digesting cortices, differ substantially not only in external morphology of the subterranean parts, but also in the diffuse tissue compartmentation in the tuber (also addressed by Goebel and Süssenguth 1924), signifying a less complex fungal regulation. Interestingly, *T. americana* (Pfeiffer 1914), also originating from the New World, much more resembles the Old World *T. rodwayi* (McLennan 1958) and *T. minutissima* (this study) with respect to root morphology and mycorrhiza.

Shepeleva et al. (2020) provide a phylogenetic tree of 40 *Thismia* spp. and an additional 12 members of Dioscoreales based on two nuclear and one mitochondrial genetic markers. The authors defined five clades, which only fragmentarily correspond with the classical subgeneric division based on morphology by Jonker (1938) and revisited by Kumar et al. (2017), the latter already pointing to discrepancies with emerging genetic information. The species investigated in the present study, except for *T. goodii* and *T. luetzelburgii*, are included in the sampling of Shepeleva et al. (2020), as well as *T. javanica* and *T. rodwayi*, about which we have anatomical information by Bernard and Ernst (1910) and McLennan (1958). The sequence named ‘*T. aseroe*’ in Shepeleva et al. (2020), however, must be attributed to *T. ornata* (shown by Dančák et al. 2020), and therefore, the detailed anatomical information given in Groom (1895) on *T. aseroe* cannot be directly linked to the phylogeny. Nevertheless, based on morphological data, indicating only marginal differences between *T. alba* (member of clade 5 in Shepeleva et al. 2020) and *T. aseroe* (Jonker 1948) as well as personal experiences of MD, MH and MS, the true *T. aseroe* certainly belongs to clade 5 in Shepeleva et al. (2020). The mycorrhiza data presented here corroborate this view.

The neotropical *T. panamensis* is only remotely related to the other species (Shepeleva et al. 2020), supported by the external and internal morphology of the underground parts as well as its mycorrhizal pattern presented here. *Thismia abei*, missing an exoepidermis, is a member of clade 1 in Shepeleva et al. (2020), being sister to all other Old World clades. *Thismia rodwayi* (McLennan 1958), affiliated to clade 2, has a single-layered exoepidermis, therein resembling *T. minutissima* (this study), which is not assigned to a particular clade, but diverges after clade 2 and is sister to clades 3 to 5 (after Shepeleva et al. 2020; alternative view in Dančák et al. 2020). Clade 3 in the phylogeny of Shepeleva et al. (2020) comprises species having coralloid root systems, including *T. brunneomitra* (this study). Finally, including those species attributed to clade 5, where there are *T. neptunis* (this study) and *T. alba*, the closest relative to *T. aseroe* (Groom 1895) with straight-growing hyphae in the mesoepidermis, the molecular phylogeny of Shepeleva et al. (2020) is in concordance with the increasing anatomical and mycorrhizal complexity of the subterranean organs, supporting the morphological progression sketched above. The only species with coralloid roots but segregated from clade 3 as its sister by Shepeleva et al. (2020) is *T. viridistriata*. Indeed, this species deviates from *T. brunneomitra*, *T. versteegii* and *T. clandestina* in keeping straight-growing hyphae in the mesoepidermis and lacking enlarged cells in the endoepidermis, both traits that are also present in *T. neptunis* and *T. aseroe* (clade 5). Possibly, the mesoepidermis developed in a common ancestor of *T. viridistriata* and clades 3 to 5. Whereas the straight-growing hyphae are preserved in *T. viridistriata* and clade 5, they became abolished in clade 3. Indeed, straight hyphae are less useful in abbreviated roots, and the mesoepidermis in *T. viridistriata* might be considered a plesiomorphic remainder among species with coralloid root systems. *Thismia brunneomitra* and *T. clandestina* (Fig. X/1 in Bernard and Ernst 1911) match in having a multilayered exoepidermis and isodiameterically enlarged endoepidermal cells, whereas *T. goodii* and *T. versteegii* (Fig. XII/1 in Bernard and Ernst 1911) both have only two layers of exoepidermis and radially enlarged cells in the endoepidermis. From an economic perspective, the reduction of functionless exoepidermis layers seems evolutionarily consistent, and because it comes along with the new feature of radially enlarged cells, we interpret both as apomorphic traits within coralloid *Thismia* spp.

The mycorrhizal structures of *T. javanica* (Janse 1896, Meyer 1909; Bernard and Ernst 1910), resembling those of *T. minutissima* and *T. rodwayi* (McLennan 1958), argue against a phylogenetic position close to clade 5, as it is stated by Shepeleva et al. (2020, see Fig. 7). In fact, the latter authors revealed discrepancies for *T. javanica* between their maximum likelihood and Bayesian analysis trees, along with low bootstrap percentage (59) and posterior probability (0.65), indicating phylogenetic uncertainty. Additionally, Severova et al. (2021) found incongruences in pollen characters among specimens assigned to *T. javanica*, leaving also the entity of the taxon questionable. Regardless, from an evolutionary perspective, the question of whether a single- or a two-layered epidermis is plesiomorphic in old-world *Thismia* spp. is open to discussion. In case the two-layered epidermis is plesiomorphic, the single-layered one of *T. abei* must be considered a reduction. The other conjecture, preferred here, is supported by Cheek et al. (2018), showing a single-layered epidermis in *Oxygyne duncanii*, a member of an early-diverging clade of Thismiaceae (Cheek et al. 2018; Shepeleva et al. 2020). Data on mycorrhizal structures especially in members around clade 4 and other species in clade 1 in Shepeleva et al. (2020) might help to elucidate this question.

## Conclusions

Mycorrhizal structures often are considered irrelevant to the degree of benefit to either side of the symbiosis, although this view may be about to change (Giesemann et al. 2020, 2021). Most reports on arbuscular mycorrhizae state, if at all, a *Paris*- or *Arum*-type AM (after Gallaud 1905) but neglect the multiple morphotypes that may occur within those categories (Imhof 2009). However, especially morphotypes in MHP, being elaborate entities of root anatomy and fungal colonization pattern including particular hyphal shapes within cells or tissue compartments, play an essential role in sustaining nutrient and carbon influx to the plant. Hence, mycorrhizal structures are subject to evolution and their traits can be tracked along a phylogeny. It is structure which allows function, and the sometimes cryptic structural diversity found among mycorrhizas can help to understand mycorrhizal functioning.

## Supplementary Information

Below is the link to the electronic supplementary material.

Supplementary file1 (DOCX 15 KB)

## Footnotes

1 The specimen (Wilks 1179) investigated in Imhof (1999), labeled as *Afrothismia winkleri*, turned out to be a new species, later described by Franke (2004) as *Afrothismia saingei* (Maas-van de Kamer and Maas 2010).
