# Supplementary material for: Mycorrhizal structures in mycoheterotrophic Thismia spp. (Thismiaceae): functional and evolutionary interpretations
Source: Mycorrhiza. 2022 Apr 14;32(3-4):269–80. doi: 10.1007/s00572-022-01076-3 (PMC9184416; doi:10.1007/s00572-022-01076-3)
Supplement: Supplementary file 1 — Supplementary file1 (DOCX 15 KB) [file 572_2022_1076_MOESM1_ESM.docx]

Supplementary Material to

Feller et al. (2022): Mycorrhizal structures in mycoheterotrophic *Thismia* spp. (Thismiaceae): functional and phylogenetic interpretations

| Species (number of specimen) | Location | Collector/Number | Date | References |
| --- | --- | --- | --- | --- |
| *Thismia abei* (Akasawa) Hatusima (5) | Japan, Island Kozu | Suetsugu s.n. | 03.07.2017 | Akasawa 1950 |
| *Thismia luetzelburgii* Goebel & Süssenguth (1) | Panama,  Colón | Maas et al. 2694 | no data | Maas et al. 1986 |
| *Thismia panamensis* (Standley) Jonker (1) | Panama, vicinity of Gamboa | Maas & Dressler 2702 | no data | Maas et al. 1986 |
| *Thismia brunneomitra* Hroneš, Kobrlová & Dančák (1) | Malaysia, Sarawak, Lawas District | Sochor, Hroneš & Dančák Bor25/19 | 30.01.2019 | Hroneš et al. 2018 |
| *Thismia goodii* Kiew (2) | Malaysia, Sarawak, Lawas District | Hroneš & Dančák 332020 | 20.02.2020 | Kiew 1999 |
| *Thismia minutissima* Dančák, Hroneš & Sochor (1) | Malaysia, Sarawak, Lawas District | Sochor, Hroneš & Dančák Bor28/19 | 30.01.2019 | Dančák et al. 2020 |
| *Thismia neptunis* Beccari (2) | Malaysia, Sarawak, Kubah | Sochor & Egertová Bor51/17 | 26.01.2017 | Sochor et al. 2018a |
| *Thismia viridistriata* Sochor, Hroneš & Dančák (1) | Malaysia, Sarawak, Kelabit Highlands | Dančák 2017/65 | 21.11.2017 | Sochor et al. 2018b |

**Supplementary Material, Table 1**: Material used in this study

References:

Akasawa Y (1950) A new species of *Glaziocharis* (Burmanniaceae). Japanese Journal of Botany 25: 193-196

Dančák M, Hroneš M, Sochor M (2020) *Thismia minutissima* (Thismiaceae), a remarkable new mycoheterotrophic species from Sarawak, Borneo. Kew Bulletin 75: 29

Hroneš M, Rejžek M, Sochor M, Svátek M, Kvasnica J,Egertová Z, Pereira J, Nilus R, Dančák M (2018) Two new species of *Thismia* subsect. Odoardoa (Thismiaceae) from Borneo. Plant Ecology and Evolution 151(1): 110-118

Kiew R (1999) *Thismia goodii* (Burmanniaceae), the blue-capped *Thismia*, a new species from Borneo. Gard. Bull. Singapore 51: 179-182

Maas PJM, Maas-van de Kamer H, Benthem Jv, Snelders HCM, Rübsamen T (1986) Burmanniaceae. Flora Neotropica Monograph 42: 1-189

Sochor M, Egertová Z, Hroneš M, Dančák M (2018a): Rediscovery of *Thismia neptunis* (Thismiaceae) after 151 years. Phytotaxa 340: 71-78

Sochor M, Hroneš M, Dančák M (2018b) New insights into variation, evolution and taxonomy of fairy lanterns (*Thismia*, Thismiaceae) with four new species from Borneo. Pl. Syst. Evol. 304(5): 699-721
